# A COSMIN-based systematic review of the measurement properties of cross-cultural versions of the orthorexia nervosa inventory

**DOI:** 10.3389/fpsyg.2026.1747648

**Published:** 2026-03-04

**Authors:** Hanqing Zhang, Chaolumen Bao

**Affiliations:** 1 The Affiliated Hospital of Inner Mongolia Medical University, Hohhot, China; 2 Faculty of Public Health, Mahidol University, Bangkok, Thailand; 3 Faculty of Social Sciences and Humanities, Mahidol University, Bangkok, Thailand

**Keywords:** COSMIN, eating disorder, instrument validation, orthorexia nervosa, psychometric properties

## Abstract

**Background:**

Orthorexia nervosa (ON) has attracted increasing attention in recent years, and the Orthorexia Nervosa Inventory (ONI) is one of the most widely used instruments for assessing ON. As research on ON expands across different cultural contexts, multiple countries have conducted translation and cultural adaptation studies of the ONI. However, the methodological quality of these translation procedures and the psychometric properties of the adapted versions have not yet been systematically synthesized and compared. Guided by the COSMIN framework, this systematic review aimed to evaluate the measurement quality of cross-culturally adapted ONI versions and to provide evidence for the standardized use of the ONI in clinical and research settings.

**Methods:**

This systematic review followed the COSMIN and PRISMA recommendations and was prospectively registered in PROSPERO (CRD420251218905). Using “Orthorexia Nervosa Inventory” and related terms, we systematically searched PubMed, Scopus, the Web of Science, Embase, and the China National Knowledge Infrastructure (CNKI) up to November 2025. We included peer-reviewed original studies published in English or Chinese that reported the translation and/or cultural adaptation of the ONI and its psychometric properties. Furthermore, two reviewers independently performed study selection and data extraction. Translation and adaptation methods were appraised using “Guidelines for the Process of Cross-Cultural Adaptation of Self-Report Measures” from Beaton et al. Measurement properties were evaluated using the quality criteria of Terwee et al. and the COSMIN Risk of Bias checklist across domains including structural validity, internal consistency, reliability, measurement error, construct validity, and responsiveness. A narrative synthesis was conducted.

**Results:**

The search yielded 280 records, of which five studies met the inclusion criteria, covering four countries and five language versions of the ONI (Chinese, Italian, Hungarian, and two Turkish versions). There was substantial heterogeneity in translation and cultural adaptation procedures. Only one Turkish version fully reported all five steps recommended by Beaton’s guidelines. Approximately 80% of the studies fulfilled the requirements for forward–backward translation and synthesis, whereas only approximately 40% reported an expert committee review and 40% reported pretesting. According to COSMIN ratings, approximately 60% of the studies were judged “very good” for structural validity, with the majority of versions supported by exploratory factor analysis (EFA) and/or confirmatory factor analysis (CFA) that broadly confirmed the intended factor structure. All five studies reported acceptable internal consistency (Cronbach’s α or *ω* for the total scale and subscales was within the recommended ranges). However, only the Chinese version provided data on test–retest reliability and measurement error/minimal detectable change (MDC), and measurement error was primarily estimated via the standard error of measurement (SEM) derived from Cronbach’s α, without interpretation against a minimal important change. Evidence for construct validity was limited: Only a subset of studies tested predefined hypotheses using correlations with related measures or known-groups comparisons, and these studies were mostly rated as “adequate” or “doubtful.” None of the included studies conducted multi-group factor analysis or differential item functioning (DIF) to assess cross-cultural validity/measurement invariance, and no study reported responsiveness or ROC-based cut-off determination.

**Conclusion:**

Existing cross-cultural versions of the ONI demonstrate generally good internal consistency and preliminary support for structural validity. Nonetheless, important gaps remain in the rigor of translation procedures, test–retest reliability, measurement error, and construct validity, with a striking absence of evidence on cross-cultural validity and responsiveness. Future research should rigorously adhere to established cross-cultural adaptation guidelines and COSMIN methodological standards, incorporate multi-group CFA to examine measurement invariance, and comprehensively assess test–retest reliability, measurement error, and responsiveness. In addition, clinically and functionally meaningful cut-off scores should be established to enhance the applicability and comparability of the ONI across diverse cultural contexts.

**Systematic review registration:**

https://www.crd.york.ac.uk/PROSPERO/view/CRD420251218905, Identifier: CRD420251218905.

## 1 Introduction

Orthorexia is a neologism derived from Greek that can be directly translated as “proper appetite” (Barthels et al., 2019). In 1997, Steven Bratman first observed that an intense focus on food and the pursuit of “extreme dietary purity” can lead to disordered eating, giving rise to detrimental and paradoxical outcomes (Bratman and Knight, 2004). Despite the growing body of research on orthorexia nervosa (ON) in recent years, there remains no consensus regarding its conceptualization or the specific criteria required for diagnosis. Although several provisional diagnostic criteria have been proposed for ON, notable discrepancies exist among them, and none have been incorporated into the Diagnostic and Statistical Manual of Mental Disorders (DSM-5) or the International Classification of Diseases (ICD-11) (Atchison and Zickgraf, 2022). This absence of formally recognized criteria renders the diagnosis of ON impossible within current clinical frameworks and further complicates the development of appropriate treatment strategies. Consequently, the status of ON as a potential mental disorder remains the subject of ongoing debate. Researchers have not yet reached a consensus on whether ON should be classified as a psychiatric condition, conceptualized as a distinct clinical syndrome, or regarded merely as an unhealthy pattern of eating behavior (Kummer et al., 2008). In a 2022 consensus meeting that convened 47 experts in eating disorders from 14 countries, orthorexia nervosa (ON) was characterized as a mental health condition that is conceptually related to the “Feeding and Eating Disorders” (F&ED) category in the DSM-5 (Donini et al., 2022). According to Donini et al. (2022), orthorexia nervosa (ON) is characterized by eating behaviors that are governed by self-imposed rigid rules. The avoidance of certain foods is not driven by adherence to religious practices, delusional beliefs, or economic constraints; instead, it is based on personal preferences, and it typically requires additional time for planning and preparing meals. These rules reflect an individual’s pursuit of self-defined “pure” or “healthy” nutrition. Foods that are excluded include processed foods, products containing genetically modified ingredients, and items treated with pesticides.

Increasing empirical evidence suggests that orthorexia nervosa (ON) may be associated with a range of adverse outcomes, most notably malnutrition and impairments in social functioning (Koven and Abry, 2015; Varga et al., 2013). Individuals exhibiting ON-related eating patterns may develop nutritional deficiencies due to substantial reductions in dietary variety. When these restrictive behaviors persist over time, they may lead to clinical manifestations similar to those observed in severe anorexia nervosa, including osteoporosis, anemia, hyponatremia, metabolic acidosis, pancytopenia, testosterone deficiency, and bradycardia (Park et al., 2011; Moroze et al., 2015; Dunn and Bratman, 2016).

In an attempt to identify reliable diagnostic criteria, researchers have developed numerous instruments for the measurement or diagnosis of orthorexia nervosa (ON).

1. The Bratman Orthorexia Test (BOT), created by Bratman and Knight (2004), has been widely used for identifying ON. The tool consists of 10 yes/no questions designed to indicate the presence of ON. However, this tool is not based on any methodology. Bratman defines the BOT as an “informal” or even a “non-existent” test (Bratman and Knight, 2004; Bratman, 2017), citing its poor clinical efficacy due to the lack of validation (Bundros et al., 2016; Dittfeld et al., 2016).
2. The ORTO-15 test is the most widely used tool for assessing ON (Valente et al., 2019; Tremelling et al., 2017). Designed by Donini et al. (2005), it has also been applied as a diagnostic tool for ON. The tool consists of 15 multiple-choice questions that explore cognitive-rational, clinical, and emotional aspects. Responses are rated on a 4-point Likert scale. While this tool is one of the most widely used measurement instruments, it has also faced several criticisms regarding its application. First, it has been argued to potentially overestimate the prevalence of orthorexia nervosa (ON) and fail to confirm the pathological characteristics associated with this condition. Second, some researchers have raised concerns about the reliability (Clifford and Blyth, 2019) and validity of the tool (Koven and Abry, 2015; Tremelling et al., 2017; Barnett et al., 2016). Finally, the ORTO-15 test has been criticized for not adhering to the latest diagnostic criteria established by Dunn and Bratman (2016).
3. The Eating Habits Questionnaire (EHQ), developed by Gleaves et al. (2013), shows strong conceptual integrity, but its factor structure has been a topic of debate. Other instruments have not gained widespread use, and their quality and validity still require further confirmation. The EHQ consists of 21 items designed to measure knowledge, behaviors, and emotions related to excessive concern regarding healthy eating.
4. The Dusseldorf Orthorexia Scale (DOS) is a tool developed in 2015 by Barthels, Meyer, and Pietrowsky in Germany (Valente et al., 2019; Barthels et al., 2015). This scale consists of 10 questions designed to measure normative eating behaviors, using a 4-point Likert scale. Currently, the only criticism of the DOS is that it appears to be unable to distinguish between patients with anorexia nervosa and those with orthorexia nervosa (Barthels et al., 2017).
5. The Orthorexia Nervosa Inventory (ONI) was developed by Oberle et al. (2021). It is a self-reported questionnaire consisting of three dimensions, namely (1) behaviors and preoccupation with healthy eating, (2) physical and psychosocial impairments, and (3) emotional distress, with a total of 24 items. The initial validation study demonstrated strong internal consistency (overall Cronbach’s α = 0.94; subscale Cronbach’s α > 0.86) and high test–retest reliability (*r* > 0.86).

In developing the ONI, multiple core dimensions of orthorexia nervosa were explicitly targeted. It not only assesses individuals’ healthy eating behaviors and associated preoccupations but also incorporates the evaluation of physical and psychosocial functional impairment and emotional distress (Niedzielski and KaŹmierczak-Wojtaś, 2021). In contrast, many earlier instruments focused primarily on specific behaviors or attitudes, resulting in a comparatively narrow scope. As the ONI includes dimensions of functional impairment and emotional distress, it offers a more comprehensive understanding of the psychopathological profile and functional impact of the condition in clinical contexts, rather than focusing solely on the frequency of symptoms or attitudinal tendencies. Consequently, it demonstrates greater utility for both clinical assessment and research applications. It has also been translated into multiple languages, including Chinese (Sun et al., 2024), Italian (Zagaria et al., 2023a), Hungarian (Fodor et al., 2025), and Turkish (Kaya et al., 2022; Turan et al., 2023).

However, no systematic review has yet been conducted to examine the published studies on these translated versions, and consequently, a comprehensive assessment of their psychometric properties is still lacking. Since levels of participation may vary across cultural contexts and each translation and cultural adaptation requires independent validation, it is important to question the robustness of the psychometric properties and the translation procedures of these adapted versions before they are used in practice. This systematic review aims to evaluate the psychometric properties of various translated and/or culturally adapted versions of the Orthorexia Nervosa Inventory (ONI) published in peer-reviewed journals. The objectives of the review are as follows: (1) to identify and summarize the psychometric properties of the ONI versions; (2) to critically assess the translation and cultural adaptation processes used for these different versions; and (3) to evaluate the methods used to assess the psychometric properties of the ONI, focusing on their validity, reliability, and cultural relevance.

## 2 Methods

The present systematic review was conducted in accordance with the COSMIN guidelines for health measurement instruments and reported following the PRISMA framework (Mokkink et al., 2016). The review protocol was registered in PROSPERO (CRD420251218905). All relevant literature published up to November 2025 was systematically reviewed.

### 2.1 Search strategy

The search strategy incorporated the term “Orthorexia Nervosa Inventory” in combination with keywords related to measurement properties, including reliability, validity, responsiveness, “minimal detectable change,” “minimal clinically important difference,” assessment tools, outcome measures, translation, and factor analysis. Relevant controlled vocabulary was also applied when appropriate (details are provided in Appendix A1). Boolean operators “OR,” “AND,” and “NOT” were used to refine the search. Searches were conducted in the following electronic databases: PubMed, Scopus, the Web of Science, Embase, and the China National Knowledge Infrastructure (CNKI).

Search strategies were adapted to the specific syntax and indexing terms of each database. No restrictions were placed on publication year, but only articles published in English or Chinese were considered. The reference lists of all included studies were also manually screened to identify additional eligible publications. The search was initially performed in October 2025 and last updated in November 2025.

### 2.2 Eligibility criteria

The inclusion criteria for the articles were as follows:

1. Studies related to the cultural adaptation and/or translation of the Orthorexia Nervosa Inventory (ONI);
2. articles written in English or Chinese and published in peer-reviewed scientific journals.

The exclusion criteria were as follows: Systematic reviews, meta-analyses, doctoral dissertations, proofreading notes or previously published materials, commentaries, editorials, and poster presentations.

To ensure adequate inter-rater reliability, the first five abstracts identified through the search were independently screened by the reviewers using the predefined inclusion criteria. Any discrepancies in screening decisions were discussed until a consensus was reached. After harmonizing the screening approach, the same reviewers proceeded to assess the full texts of all studies that met the initial eligibility criteria. In addition, the reference lists of all included articles were manually examined to identify any additional studies that might meet the inclusion criteria but were not captured during the database search (Abid et al., 2023).

The titles and abstracts were imported into the Covidence software platform,^1^ which is an online tool designed to support systematic review workflows. Two authors (HQ and XZ) independently screened the titles and abstracts for the initial selection.

### 2.3 Critical appraisal of the articles

1. The quality of the translation procedures used in the included studies was appraised in accordance with the *Guidelines for the Process of Cross-Cultural Adaptation of Self-Report Measures* (Beaton et al., 2000). These guidelines delineate five key stages in the adaptation process, namely forward translation, synthesis of translations, back-translation, expert committee review, and pretesting. Each component was evaluated using a four-level rating system: “+” indicating adequate performance, “−” indicating inadequate performance, “0” signifying that no information was provided, and “?” denoting insufficient clarity. Comprehensive descriptions of these procedures and the corresponding rating criteria are provided in the study conducted by Costa et al. (2009).
2. The clinimetric properties were appraised using the *Quality Criteria for Psychometric Properties of Health Status Questionnaires* (Terwee et al., 2007), with the assessment limited to those items relevant to cross-cultural adaptation. Since content validity and interpretability are related only to the original development of an instrument, and criterion validity can only be evaluated in the presence of an established gold standard—which is absent in the field of Orthorexia Nervosa—these three criteria were deemed not applicable for this review. The Terwee criteria comprise a checklist that considers both the methodological quality of clinimetric testing and the outcomes derived from it. This approach differs from tools designed to assess the methodological rigor of clinical trials. Given that various clinimetric properties represent distinct constructs, the criteria do not yield a single composite score. Instead, the findings are presented in a table to offer a clear and comprehensive summary of the quality of testing procedures and the resulting clinimetric evidence. This method has been adopted in previous systematic reviews of related questionnaires (Costa et al., 2009; Terwee et al., 2007).
3. The methodological quality of the included studies was assessed using the COSMIN 2018 methodology (Prinsen et al., 2018) and the *Risk of Bias checklist* (Mokkink et al., 2018). This framework, designed to guide optimal measurement instrument selection, evaluates 10 domains covering instrument development standards and 9 key psychometric properties, namely content validity, structural validity, internal consistency, cross-cultural validity/measurement invariance, reliability, measurement error, criterion validity, hypothesis testing for construct validity, and responsiveness. Each psychometric property was rated on a four-point scale (very good, adequate, doubtful, and inadequate) according to the COSMIN group’s established classification system, which provided the evaluation framework for this review (Prinsen et al., 2018).

Based on this framework, two reviewers independently evaluated the data extracted from the included studies using three assessment tools: The *Guidelines for the Process of Cross-Cultural Adaptation of Self-Report Measures*, the *Quality Criteria for Measurement Properties of Health Status Questionnaires*, and the COSMIN Risk of Bias checklist.

### 2.4 Extraction and synthesis

The following data were extracted from the selected articles and organized into two tables. One table contained variables related to the study characteristics: Publication date (year), instrument name, country, survey participants, sample size (number of individuals/cases), number of dimensions/items, Cronbach’s α/McDonald’s *ω*, and test–retest time. The other table summarized the psychometric characteristics.

A consensus-based process was employed for data extraction using Covidence software. Moreover, two reviewers (HQ and XZ) performed the extraction independently, followed by an iterative process of comparison and discussion. Persistent disagreements were resolved through third-party arbitration.

## 3 Results

**Figure 1:**
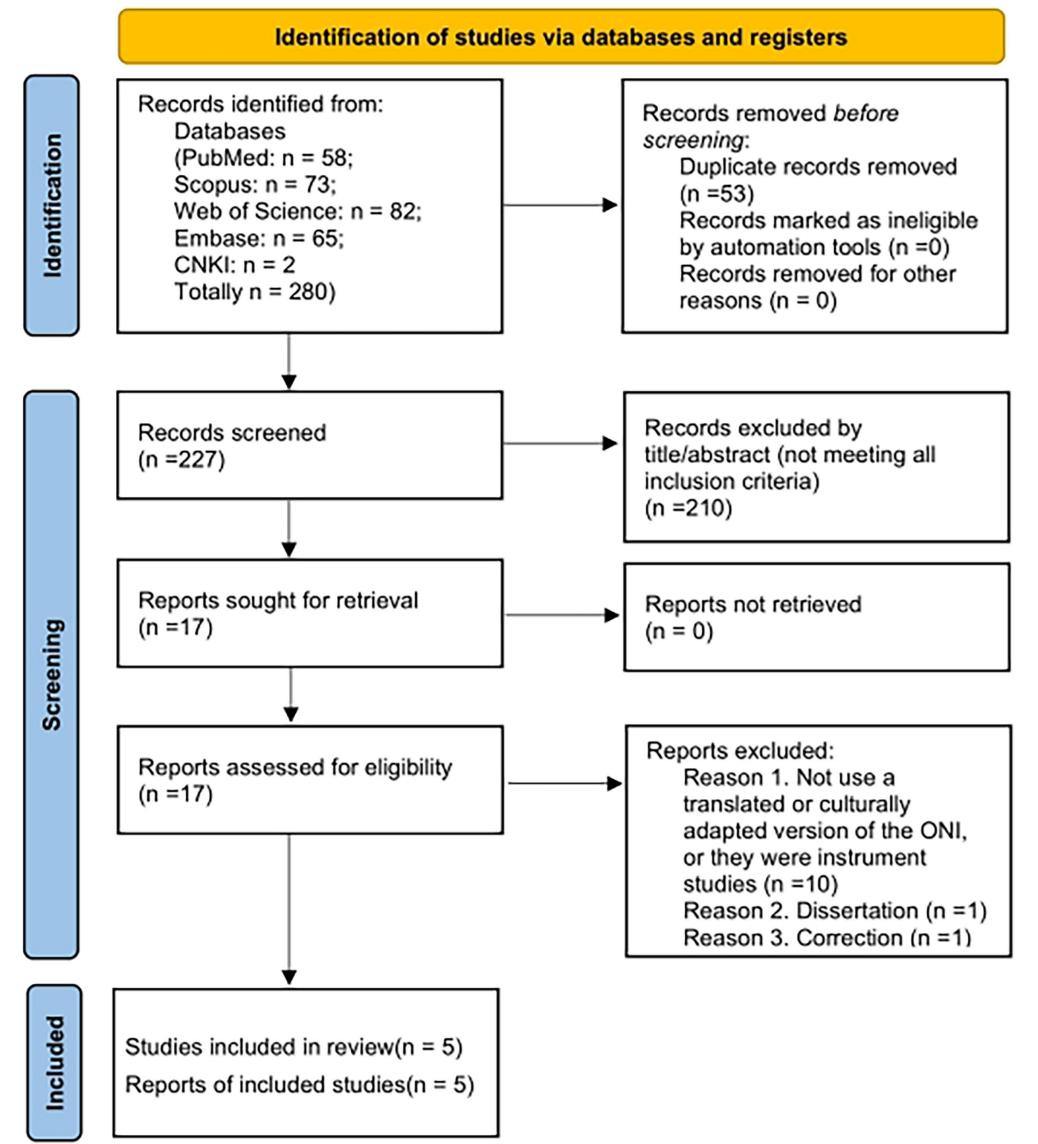
PRISMA flow diagram.

The search strategy yielded 280 records (PubMed: *n* = 58; Scopus: *n* = 73; Web of Science: *n* = 82; Embase: *n* = 65; CNKI: *n* = 2). After removing duplicates (*n* = 53), 227 records remained for title and abstract screening, of which 210 were excluded. A total of 17 full-text articles were assessed for eligibility, and 11 of these were subsequently excluded for failing to use a translated or culturally adapted version of the ONI or for other ineligibility reasons. Specifically, the exclusion included instrument studies (Toğuç and Hökelek, 2025; Duradoni et al., 2025; Yildirim et al., 2025; Unal and Kocatepe Avcı, 2024; Ephrem et al., 2024; Yılmaz et al., 2024; Liu et al., 2024; Tabri et al., 2022; Brunett and Oberle, 2022; Noebel et al., 2022) (*n* = 10), a dissertation (Li, 2023) (*n* = 1), and an updated corrigendum to a previously included study (Zagaria et al., 2023b) (*n* = 1). A total of five studies met the inclusion criteria and were retained for analysis (Figure 1). All five included studies employed a cross-sectional design and relied on voluntary participant recruitment. The extracted data from these studies are summarized in Table 1. Model fit indices are presented in Table 2. Table 3 presents the evaluation of the translation procedures following the *Guidelines for the Process of Cross-Cultural Adaptation of Self-Report Measures*, while the COSMIN Risk of Bias checklist assessments are reported in Table 4. Table 5 presents the clinimetric testing of the ONI versions based on the Quality Criteria for Psychometric Properties of Health Status Questionnaire. The five studies included adaptations in the following languages and countries: Chinese (Sun et al., 2024), Italian (Zagaria et al., 2023a), Hungarian (Fodor et al., 2025), and Turkish (Kaya et al., 2022; Turan et al., 2023).

**Table 1:** General characteristics of the included literature.

Title	Authors and year	Scale	Language	Characteristics of the sample	Sample size	Dimensions/items	Psychometric properties Cronbach’s α/McDonald’s *ω*	Test–retest days
Cross-cultural adaptation and psychometric properties of the Chinese version of the Orthorexia Nervosa Inventory	Sun et al. (2024)	Chinese version of the Orthorexia Nervosa Inventory (C-ONI)	Chinese	Adults	717	3 dimensions/24 items	Cronbach’s α (total: 0.956, Behaviors: 0.894, Impairments: 0.933, Emotions: 0.848) McDonald’s ω (total: 0.957, Behaviors: 0.895, Impairments: 0.934, Emotions: 0.854)	14
Cross-cultural adaptation and psychometric properties of the Italian version of the Orthorexia Nervosa Inventory (ONI)	Zagaria et al. (2023a)	I-ONI (Italian version of ONI)	Italian	Community sample	879	3 dimensions/24 items	Cronbach’s α total: 0.948, Behaviors: 0.885; Impairments: 0.884; Emotions: 0.913	NA
Beyond healthy eating: introducing ONI-Hu, the Hungarian version of the Orthorexia Nervosa Inventory	Fodor et al. (2025)	ONI-Hu (Hungarian version of the Orthorexia Nervosa Inventory)	Hungarian	Adults	922	3 dimensions/24 items	Cronbach’s α (total: 0.91, Behaviors: 0.88, Impairments: 0.80, Emotions: 0.83) McDonald’s ω (total: 0.91, Behaviors: 0.89, Impairments: 0.81, Emotions: 0.85)	NA
Psychometric properties of the Turkish Orthorexia Nervosa Inventory in a clinical adolescent sample	Turan et al. (2023)	Turkish Orthorexia Nervosa Inventory (ONI-TR)	Turkish	Adolescents aged 12–18 years who applied to the Department of Child and Adolescents Psychiatry	266	3 dimensions/24 items	Cronbach’s α total: 0.92, Behaviors: 0.84; Impairments: 0.84; Emotions: 0.83	NA
Validation of the Turkish version of the Orthorexia Nervosa Inventory (ONI) in an adult population: its association with psychometric properties	Kaya et al. (2022)	Turkish version of the ONI	Turkish	General adult population	710	3 dimensions/24 items	Cronbach’s α total: 0.91, Behaviors: 0.82; Impairments: 0.84; Emotions: 0.81	NA

**Table 2:** Model fit indices.

Translation language	χ^2^/df	RMSEA	CFI	TLI	SRMR
Chinese	2.42	0.092	0.994	NR	0.043
Italian	4.26	0.041–0.061 (ESEM provided the best fit)	0.981 (ESEM)	0.972 (ESEM)	0.033 (ESEM)
Hungarian	3.73	0.067	0.932	0.923	NR
Turkish (Turan, B.)	1.89	0.058	0.92	0.909	0.033
Turkish (Kaya, S.)	5.65	0.08	0.94	0.93	0.07

NR, Not Report.

*Calculated and added χ^2^/df for the Hungarian version (929/249 = 3.73).

**Table 3:** Cross-cultural adaptations of the MPQ that used the translation-based approach relate to the Guidelines for the Process of Cross-Cultural Adaptation of Self-Report Measures.

Translation language (country)	Author (year of publication)	Translation	Synthesis	Back translation	Expert committee	Pretesting
Chinese	2025	+	+	+	+	0
Italian	2023	+	+	+	0	+
Hungarian	2025	−	+	−	0	0
Turkish (Turan, B.)	2023	+	+	+	0	0
Turkish (Kaya, S.)	2022	+	+	−	+	+

Positive ranking (+).

Negative ranking (−).

No information available (0).

Unclear (?).

**Table 4:** Critical appraisal of the methodology used to translate the ONI using Guidelines for the Process of Cross-Cultural Adaptation of Self-Report Measures.

Translation language	Box 3 Structural validity	Box 4 Internal consistency	Box 5 Cross-cultural validity/measurement invariance	Box 6 Reliability	Box 7 Measurement error	Box 9 Hypotheses testing for construct validity
Chinese	++	++	NA	?	?	?
Italian	++	++	++	NA	NA	++
Hungarian	++	++	NA	NA	NA	?
Turkish (Turan, B.)	?	++	NA	NA	NA	+
Turkish (Kaya, S.)	?	++	NA	NA	NA	++

Very good (++).

Adequate (+).

Doubtful (?).

Inadequate (−).

NA, Not Access.

*COSMIN RoB Boxes 1, 2, 8, and 10 were not evaluated in this review because the included studies did not report these aspects (i.e., PROM development, content validity, cross-cultural validity/measurement invariance, and responsiveness). Therefore, these boxes are not presented in the table.

**Table 5:** Clinimetric testing of the ONI versions relate to Quality Criteria for Psychometric Properties of Health Status Questionnaire.

Title	Reliability			Validity				Responsiveness
	Internal consistency	Measurement error	Reliability	Content validity	Construct validity			
					Cross-cultural validity	Structural validity	Hypothesis testing	
Chinese	+	?	+	+	?	+	+	0
Italian	+	0	0	?	?	?	+	0
Hungarian	+	0	0	?	?	?	+	0
Turkish (Turan, B.)	+	0	0	?	?	0	+	0
Turkish (Kaya, S.)	+	0	0	?	?	0	−	0

Positive rating (+).

Doubtful design or method (?).

Negative rating (−).

No information available (0).

### 3.1 Translations

To cross-culturally adapt a self-administered questionnaire for assessing health status to be suitable for a new country, culture, and/or language, it is necessary to apply a specific set of methods to ensure that the original version and the target version are equivalent. It is now recognized that if an instrument is to be used across different cultures, the items should not only be translated accurately at the linguistic level but also need to be culturally adapted to maintain the content validity of the instrument at the conceptual level across cultures (Beaton et al., 2000). Among the five steps recommended in the Guidelines (Beaton et al., 2000) for the Process of Cross-Cultural Adaptation of Self-Report Measures (Beaton et al., 2000)—(1) translation, (2) synthesis, (3) back-translation, (4) expert committee review, and (5) pretesting—only the Turkish (Kaya et al., 2022) version completed the full adaptation process.

1. 80% (*n* = 4) of the studies (Sun et al., 2024; Zagaria et al., 2023a; Kaya et al., 2022; Turan et al., 2023) met the standards for the translation step, while the Hungarian (Fodor et al., 2025) study reported that translation was performed by a single translator.
2. All studies completed the synthesis step (*n* = 5).
3. Among the included studies, only one reported that back-translation was conducted by a single translator (Fodor et al., 2025). In addition, 80% (*n* = 4) of the studies adequately completed this step.
4. Only 40% (*n* = 2) of the studies (Sun et al., 2024; Kaya et al., 2022) underwent an expert committee review.
5. Only 40% (*n* = 2) of the studies (Zagaria et al., 2023a; Kaya et al., 2022) conducted pretesting.

### 3.2 Cosmin risk of BIAS checklist

In this systematic review, the COSMIN Risk of Bias checklist was applied to evaluate the methodological quality of the included studies.

#### 3.2.1 Structural validity

Definition: The degree to which the scores of a health-related patient-reported outcome (HR-PRO) instrument effectively reflect the dimensionality of the construct to be measured (Mokkink et al., 2010).

A total of 60% (*n* = 3) of the articles were rated as very good (++). The Chinese (Sun et al., 2024), Italian (Zagaria et al., 2023a), and Hungarian (Fodor et al., 2025) versions of the Orthorexia Nervosa Inventory met the COSMIN requirements for classical test theory. Specifically, confirmatory factor analysis (CFA) was conducted. The sample size met the criterion of being at least seven times the number of items and exceeded 100 participants, and no statistical methods were applied inappropriately. The two Turkish (Kaya et al., 2022; Turan et al., 2023) translations of the ONI were classified as doubtful (?). When the first study (Turan et al., 2023) translated the instrument for the first time and applied it to a clinical population that differed substantially from the original sample, it relied solely on CFA to test the hypothesized factor structure, without any empirical exploration. This provides no independent evidence for the applicability of the *a priori* model in the new population. In addition, the study did not report the specific estimation method used for the CFA. The second manuscript (Kaya et al., 2022) specified that the CFA was conducted using maximum likelihood (ML); however, since the ONI items are 4-point Likert-type ordinal indicators, an estimator designed for ordinal data (e.g., WLSMV) is generally recommended. Otherwise, parameter estimates, standard errors, and global fit indices may be biased or affected (Park, 2023).

#### 3.2.2 Internal consistency

Definition: The degree of interrelatedness among the items (Mokkink et al., 2010).

Five studies (Sun et al., 2024; Zagaria et al., 2023a; Fodor et al., 2025; Kaya et al., 2022; Turan et al., 2023) reported internal consistency assessments (Cronbach’s α coefficient). Internal consistency was calculated for each unidimensional scale or subscale, and Cronbach’s α or *Ω* coefficients were reported. They were rated as very good (++).

#### 3.2.3 Reliability

Definition: The degree to which the measurement is free from measurement error (Mokkink et al., 2010).

Only the Chinese translation of the ONI (Sun et al., 2024) reported test–retest reliability, but it did not provide sufficient details and was deemed doubtful (?). The other studies did not assess test–retest reliability.

#### 3.2.4 Measurement error and minimal detectable change

Definition: The systematic and random error of a patient’s score that is not attributed to true changes in the construct to be measured (Mokkink et al., 2010).

Only the Chinese translation of the ONI (Sun et al., 2024) reported measurement error and the minimal detectable change (MDC). However, only the standard error of measurement (SEM) calculated based on Cronbach’s α was reported, and it was judged as inadequate (−). The remaining studies did not report any information related to measurement error or the MDC.

#### 3.2.5 Construct validity

Definition: The degree to which the scores of an HR-PRO instrument are consistent with hypotheses (e.g., with regard to internal relationships, relationships to scores of other instruments, or differences between relevant groups) based on the assumption that the HR-PRO instrument validly measures the construct to be measured (Mokkink et al., 2010).

In the construct validity domain, the Italian (Zagaria et al., 2023a) and Hungarian (Fodor et al., 2025) versions of the ONI were rated as very good (++) as they (i) used clearly defined comparator instruments, (ii) employed comparator measures with adequate measurement properties, and (iii) applied appropriate statistical methods. In contrast, the Chinese version (Sun et al., 2024) and both Turkish versions (Kaya et al., 2022; Turan et al., 2023) were rated as doubtful (?) because the between-subgroup comparisons (i.e., discriminative/known-groups validity) relied on suboptimal statistical approaches and other minor methodological shortcomings (e.g., presenting data only in comparison to another structural tool).

#### 3.2.6 Cross-cultural validity/measurement invariance

Definition: The degree to which the performance of the items of a translated or culturally adapted HR-PRO instrument is an adequate reflection of the performance of the items in the original version of the HR-PRO instrument (Mokkink et al., 2010).

Among the included studies, only the Italian version (Zagaria et al., 2023a) assessed measurement invariance, and it was rated as very good. A multi-group exploratory structural equation modeling framework was employed to examine invariance, meeting the COSMIN requirements for sample size. Apart from the grouping variable, the samples exhibited similar relevant characteristics, and no other significant methodological flaws were identified.

### 3.3 Evaluation of the ONI versions based on Terwee’s quality criteria

Using the quality criteria proposed by Terwee et al. (2007), this review identified clear heterogeneity in the measurement properties of existing ONI translations. All versions demonstrated adequate internal consistency; however, the reporting of measurement error, test–retest reliability, and responsiveness was incomplete or absent, resulting in “No information available (0)” ratings for these domains and revealing substantial methodological limitations for longitudinal or evaluative use.

For validity, across the five included studies, none achieved a “positive rating +” for content validity, as no study provided evidence that the target population considered all questionnaire items relevant or that the questionnaire was complete. Turkish version (Kaya et al., 2022): Indeterminate rating—conducted a small target-population pretest (*n* = 10) focused on clarity but did not report whether the target population judged all items relevant or considered the questionnaire complete. Italian version (Zagaria et al., 2023a): Indeterminate rating—conducted a target-population pretest (*n* = 20) to ensure that items were understandable but provided no evidence that participants evaluated item relevance and comprehensiveness. Chinese version (Sun et al., 2024): Indeterminate rating—performed a Delphi procedure with CVI indices, but target population involvement in judging item relevance and questionnaire completeness was not reported. Turkish version (Turan et al., 2023): Indeterminate rating—translation and adaptation were reviewed by clinicians, but there was no description of target population involvement in assessing item relevance or questionnaire completeness. Hungarian version (Fodor et al., 2025): Indeterminate rating—translation, back-translation, and author review were reported, but there was no report of target population involvement to confirm item relevance or scale completeness.

All five studies confirmed the original three-factor structure; however, four of them did not conduct measurement invariance analysis (at least metric/scalar) via multi-group CFA or differential item functioning (DIF) analysis. Consequently, a doubtful design or method rating (?) was assigned. In the Italian version of the ONI, the scale exhibited factorial invariance between male and female groups, making it less likely to cause confusion due to measurement bias when comparing scores across sexes. Consequently, a positive rating (+) was assigned (Zagaria et al., 2023a). In the section on structural validity, only the Chinese version of the ONI met the criterion that factors should explain at least 50% of the variance. For the Italian version of the ONI, explained variance was not mentioned, while the remaining three studies did not report this outcome. In the hypothesis testing section, the four studies that met the criterion—correlation with an instrument measuring the same construct ≥ 0.50 or at least 75% of the results in accordance with the hypotheses and demonstrated higher correlations with related constructs than with unrelated constructs were rated as positive (+). The Turkish version (Kaya et al., 2022) of the ONI received a negative rating (−) because its correlation with an instrument measuring the same construct was <0.50.

## 4 Discussion

This systematic review synthesized evidence from existing translated and culturally adapted versions of the ONI, examining their reported psychometric properties. Among the five available versions across four languages, a range of translation and adaptation procedures was applied. Cross-cultural adaptation is defined as the degree to which the items of a translated or culturally adapted instrument adequately reflect the performance of the items in the original version (Prinsen et al., 2018). This review indicates substantial variation in the rigor with which translation-based cross-cultural/linguistic adaptation (CCA) procedures were implemented across the five studies. Overall, the Chinese version (Sun et al., 2024) completed key steps, including forward translation, synthesis, back-translation, and expert committee review, but it did not conduct (or did not report) pretesting. The Italian version (Zagaria et al., 2023a) and the Turkish version (Kaya et al., 2022) both included pretesting; however, one lacked an expert committee review and the other omitted back-translation. The Turkish version (Turan et al., 2023) completed the translation–back-translation chain but did not report an expert committee review or pretesting. The Hungarian version (Fodor et al., 2025) reported only synthesis, with other essential steps missing or not described. Collectively, while several studies covered most core procedures, incomplete reporting and/or omission of pretesting, expert review, and back-translation remained common, weakening the evidence for semantic and conceptual equivalence and limiting conclusions about the comprehensibility and contextual appropriateness of the translated versions. Poor translation can result in a tool that is not equivalent to the original questionnaire. This lack of equivalence limits the comparability of responses between different language or cultural groups (Herdman et al., 1998).

Only one study (Sun et al., 2024) assessed test–retest reliability, making meaningful comparisons of the stability of the ONI across versions impossible. Future research should prioritize rigorous evaluation of reproducibility to strengthen the evidence base for its use. The Italian (Zagaria et al., 2023a) ONI demonstrated gender measurement invariance, enhancing its interpretability and supporting valid cross-gender comparisons, thereby representing a methodological strength not yet achieved by other language versions. The MDC serves as a “universal language” for cross-cultural comparisons: To compare the sensitivity of a scale across different cultural versions or to determine whether score differences between cultures are real, it is essential to rely on reliable MDC values specific to each version (Seamon et al., 2022). The absence of MDC deprives such comparisons of an objective benchmark. Conversely, the MIC addresses whether a change is “important” (Terwee et al., 2021). The lack of MIC means we cannot judge whether a “real” change is also “meaningful.” However, none of the included studies assessed this essential part of the research, representing a major limitation. This omission likely reflects the predominance of cross-sectional validation designs, coupled with a lack of repeated measurements and suitable external anchors. This collectively constitutes a major limitation: The existing versions of the ONI cannot be reliably used to assess individual changes over time (such as intervention effects), greatly limiting their application value in longitudinal studies and clinical monitoring. Furthermore, the ongoing debate regarding the conceptual boundaries of orthorexia nervosa and the absence of a diagnostic gold standard further exacerbate this challenge.

### 4.1 Recommendations for future research

1. The process of translation and cultural adaptation requires standardization. Among the five included studies, only 20% (*n* = 1; Kaya et al., 2022) fully adhered to the five-step Beaton framework (translation, synthesis, back-translation, expert committee review, and pretesting). The remaining studies did not implement the complete translation process. Future research should rigorously follow internationally recognized guidelines for translation and adaptation, comprehensively document each procedural step, and place particular emphasis on strengthening expert committee review and pretesting with the target population to ensure semantic, conceptual, and functional equivalence. Given the diversity of dietary cultures, incorporating locally relevant food items through rigorous methodological approaches in cross-cultural studies can enhance the measurement of the intended concepts.
2. Given the diversity of dietary practices across cultures (Lee et al., 2017; Lipoeto et al., 2013; Pries et al., 2025), future cross-cultural ONI research may benefit from using Delphi panels to develop region-specific items that better capture culturally distinct expressions of orthorexia behaviors. Sociocultural differences appear to be part of the discussion of many validated ON tools (Carpita et al., 2024). Many societies have deeply ingrained cultural ties to food, which complicate the distinction between a genuine pursuit of healthy dietary practices and an obsessive fixation, which is often classified as compulsive eating behavior. Indeed, while certain cultural traditions have long emphasized wholesome nutrition as a cornerstone of daily living, other societies may hold significantly divergent views on this subject. For instance, a study conducted in China by He et al. (2019) reported a prevalence rate of orthorexia nervosa (ON) of 7.8%, as assessed using the Düsseldorf Orthorexia Scale (DOS). The authors further suggested that the risk of ON in this population might be elevated, given the cultural context of a longstanding tradition that prioritizes healthy eating. Therefore, the Delphi method represents a pivotal phase in the adaptation process, during which modification and/or supplementation of items to enhance their cultural relevance and contextual appropriateness for the target population is imperative. Such additions could enhance the instrument’s cultural relevance and improve its sensitivity in different populations.
3. In future research, ROC curve analysis could be considered as an additional, application-oriented step when the ONI (or its adaptations) is intended for screening purposes. ROC methods can help evaluate discriminative performance between predefined groups and derive candidate cut-off values by balancing sensitivity and specificity; the AUC may also facilitate comparisons across versions and samples. However, ROC evidence was not reported in the studies included in this review and, therefore, should not be interpreted as supporting the performance of any translation. We have accordingly presented ROC analysis here only as a forward-looking recommendation, to be conducted in well-designed clinical or high-risk samples with an appropriate external criterion (e.g., diagnostic interview or validated comparator) (Hajian-Tilaki, 2013).

### 4.2 Limitations of the review

A central focus of this systematic review was the rigorous application of internationally recommended guidelines for the translation, cross-cultural adaptation, and quality assessment of the psychometric properties of the included instruments. The methodological approach prioritized the evaluation of these processes against established standards to ensure the validity and reliability of findings across different linguistic and cultural contexts. However, a notable limitation was imposed on the scope of the review due to the linguistic expertise of the research team. Consequently, the literature search and subsequent inclusion of articles were restricted to publications in English or Chinese. This language restriction may have introduced selection bias, potentially omitting relevant studies published in other languages and limiting the comprehensiveness of the evidence on the cross-cultural validity of the measures.

## 5 Conclusion

This systematic review identified five translated and/or culturally adapted versions of the Orthorexia Nervosa Inventory (ONI), covering four countries and five distinct languages. Heterogeneity was observed in both the methodological rigor of the translation procedures and the comprehensiveness of the psychometric property evaluations. Several of the included studies did not meet key methodological standards specified in the COSMIN Risk of Bias checklist, particularly concerning essential stages of the cross-cultural adaptation process and the assessment of critical measurement properties. A notable finding is the insufficient or entirely absent evaluation of fundamental psychometric domains—most prominently, cross-cultural validity and measurement invariance—across all adapted versions. Furthermore, given that the majority of versions lack empirical evidence for measurement error (e.g., minimal detectable change) and responsiveness, their current use is not recommended for clinical diagnostic purposes or for evaluating individual-level changes over time, such as in treatment outcome studies. Consequently, it is imperative to conduct further methodologically rigorous studies to address these evidence gaps and to provide robust empirical support for the currently under-evaluated measurement properties. As the ONI gains increasing prominence in both research and clinical contexts, future translations and culturally sensitive adaptations must adhere to established guidelines to ensure the instrument’s broader applicability and to establish measurement equivalence across diverse linguistic and cultural populations.

## Data Availability

The original contributions presented in the study are included in the article/supplementary material, further inquiries can be directed to the corresponding author.

## Footnotes

1
https://www.covidence.org/
