# Supplementary material for: A COSMIN-based systematic review of the measurement properties of cross-cultural versions of the orthorexia nervosa inventory
Source: Front Psychol. 2026 Mar 4;17:1747648. doi: 10.3389/fpsyg.2026.1747648 (PMC12996259; doi:10.3389/fpsyg.2026.1747648)
Supplement: Supplementary file 1 [file Supplementary_file_1.docx]

**S2 Appendix :  Databases search strategy :**

| **Concept 1:**  Orthorexia Nervosa and Orthorexia Nervosa Inventory OR ONI | | | | | | | | | | |  |
| --- | --- | --- | --- | --- | --- | --- | --- | --- | --- | --- | --- |
| **Free vocabulary** | (OR) | **Controlled vocabulary**(thesaurus of databases) | | | | | | | | | |
| « Orthorexia » or  « 完美食欲症 » or  « Orthorexia Nervosa » and  « Orthorexia Nervosa Inventory »  OR « ONI » |  | PubMed | (OR) | Scopus | (OR) | EMBASE (ELSEVIER) | (OR) | China National Knowledge Infrastructure (CNKI). | (OR) | WEB OF  SCIENCE Core Collection (CLARIVATE) | |
|  |  | 58 |  | 73 |  | 65 |  | 2 |  | 82 | |



**AND**

| **Concept 2:** Psychometric properties | | | | | | | | | | |
| --- | --- | --- | --- | --- | --- | --- | --- | --- | --- | --- |
| **Free vocabulary** | (OR) | **Controlled vocabulary**(thesaurus of databases) | | | | | | | | |
| measurement OR  reliability OR  validity OR  responsiveness OR  “Minimal detectable change” OR  “Minimal clinically important difference” OR  tools OR assessment OR measure* OR  “Outcome measure”  OR translation OR “factor analysis” OR validation |  | PubMed | (OR) | Scopus | (OR) | EMBASE | (OR) | China National Knowledge Infrastructure (CNKI). | (OR) | WEB OF  SCIENCE Core Collection (CLARIVATE) |
|  |  | “Measurement Issues and Assessments” OR Validity OR Reliability OR   “Outcome Assessment” |  | “Reproducibility of Results” OR  “Minimal Clinically Important Difference” OR  “Outcome Assessment, Health Care” OR  “Patient Outcome Assessment” |  | ‘Measurement’ OR ‘Reliability’ OR ‘Responsiveness’ OR ‘Minimum detectable change’ OR ‘Minimal clinically important difference’ OR ‘Tool’ OR ‘Tool use’ OR ‘Translation’ OR ‘Factor analysis’ OR ‘Validation’ OR ‘Assessment of humans’ OR ‘Outcome assessment’ OR ‘Validation process’ OR ‘validation study’ |  | ‘’Consistency (Measurement)’’/OR ‘’Error of Measurement’’/ OR Variability Measurement/ OR Statistical Measurement/ OR Measurement/ OR Test-Retest Reliability/ OR Interrater Reliability/ OR Test Reliability/ OR Statistical Reliability/ OR Factorial Validity/ OR Discriminant Validity/ OR Construct Validity/ OR Test Validity/ OR Concurrent Validity/ OR Clinical Validity/ OR Statistical Validity/ OR Internal Validity/ Or Cross Cultural Validity/ OR Content Validity/ OR External Validity/ OR Incremental Validity/ OR Predictive Validity/ OR Discriminative Validity/ OR Convergent Validity/ OR Face Validity/ OR Criterion Validity/ OR Psychometrics/ OR Test Responsiveness/ OR Rating scales/ OR Questionnaires/ OR Foreign Language Translation/ OR Test Construction/ OR ‘’Tool Use’’/ OR Screening Tests OR Self-Report/ OR Patient Reported Outcome Measures/ OR Factor Analysis/ OR Cross Cultural Differences/ |  | TS= (measurement OR  reliability OR  validity OR  responsiveness OR  “minimal detectable change” OR  “minimal clinically important difference” OR  tools OR assessment OR measure* OR “outcome measure”  OR translation OR “factor analysis” OR validation) |
